# The inhibition of assembly of HIV-1 virus-like particles by 3-*O*-(3',3'-dimethylsuccinyl) betulinic acid (DSB) is counteracted by Vif and requires its Zinc-binding domain

**DOI:** 10.1186/1743-422X-5-162

**Published:** 2008-12-23

**Authors:** Sandrina DaFonseca, Pascale Coric, Bernard Gay, Saw See Hong, Serge Bouaziz, Pierre Boulanger

**Affiliations:** 1 Université de Lyon I – Claude Bernard, Faculté de Médecine Laënnec, Laboratoire de Virologie & Pathologie Humaine, CNRS FRE-3011, 69372 Lyon Cedex 08, France; 2 Université de Paris VII – René Descartes, UFR des Sciences Pharmaceutiques et Biologiques, Unité de Pharmacologie Chimique et Génétique, INSERM U-640 and CNRS UMR-8151, 75006 Paris, France; 3 Universités de Montpellier I et II, Centre d'Etudes d'Agents Pathogènes et Biotechnologies pour la santé, CNRS UMR-5236, Institut de Biologie, 4, Boulevard Henri IV, 34965 Montpellier Cedex 02, France; 4 Laboratoire de Virologie Médicale, Centre de Biologie et Pathologie Est, Hospices Civils de Lyon, 59, Boulevard Pinel, 69677 Bron Cedex, France

## Abstract

**Background:**

DSB, the 3-*O*-(3',3'dimethylsuccinyl) derivative of betulinic acid, blocks the last step of protease-mediated processing of HIV-1 Gag precursor (Pr55Gag), which leads to immature, noninfectious virions. When administered to Pr55Gag-expressing insect cells (Sf9), DSB inhibits the assembly and budding of membrane-enveloped virus-like particles (VLP). In order to explore the possibility that viral factors could modulate the susceptibility to DSB of the VLP assembly process, several viral proteins were coexpressed individually with Pr55Gag in DSB-treated cells, and VLP yields assayed in the extracellular medium.

**Results:**

Wild-type Vif (Vif^wt^) restored the VLP production in DSB-treated cells to levels observed in control, untreated cells. DSB-counteracting effect was also observed with Vif mutants defective in encapsidation into VLP, suggesting that packaging and anti-DSB effect were separate functions in Vif. The anti-DSB effect was abolished for VifC133S and VifS116V, two mutants which lacked the zinc binding domain (ZBD) formed by the four H^108^C^114^C^133^H^139 ^coordinates with a Zn atom. Electron microscopic analysis of cells coexpressing Pr55Gag and Vif^wt ^showed that a large proportion of VLP budded into cytoplasmic vesicles and were released from Sf9 cells by exocytosis. However, in the presence of mutant VifC133S or VifS116V, most of the VLP assembled and budded at the plasma membrane, as in control cells expressing Pr55Gag alone.

**Conclusion:**

The function of HIV-1 Vif protein which negated the DSB inhibition of VLP assembly was independent of its packaging capability, but depended on the integrity of ZBD. In the presence of Vif^wt^, but not with ZBD mutants VifC133S and VifS116V, VLP were redirected to a vesicular compartment and egressed via the exocytic pathway.

## Introduction

The 3-*O*-(3',3'-dimethylsuccinyl)-betulinic acid (or YK-FH312 [1], or PA-457 [2], or Bevirimat™ [3,4]), has been used as an antiviral which blocks HIV-1 replication via its inhibitory activity on Gag polyprotein maturation [2,5-8]. DSB differs from conventional protease (PR) inhibitors in that it does not bind to PR, but interferes with the PR-mediated Gag processing. The ultimate cleavage of the C-terminal capsid domain CAp25 into CAp24 + SP1 is required for production of fully infectious virions [9]. DSB blocks this step, and decreases or abolishes virus infectivity [2,4,6,10]. Several lines of evidence indicate that the CA-SP1 junction is the preferred target of DSB in HIV-1 Gag precursor [3,4,8,11]. Although there is no available structural data on DSB-Gag complex which could explain its inhibitory activity at the molecular level, data from *in vitro *experiments [12], as well as the encapsidation of DSB in equimolar ratio to Gag *in vivo *[13], suggested that the mechanism of inhibitory activity of DSB results from the direct binding of DSB to the Gag polyprotein, or/and to a transient Gag structural intermediate which occurs during virus assembly.

The latter observation incited us to study the possible effect of DSB on assembly of recombinant HIV-1 Gag precursor (Pr55Gag) expressed in heterologous, eukaryotic system. We observed a dose-dependent negative effect of DSB on the process of assembly and release of HIV-1 VLP from recombinant baculovirus AcMNPV-Pr55Gag-infected cells [14]. This effect was not due to a block in Gag synthesis, and was independent of the N-myristoylation of Pr55Gag and its plasma membrane addressing. It did not depend on the presence of the p6 domain at the C-terminus of Gag. The same effect was observed with the Gag precursor of SIVmac (Pr57Gag^SIV^), although at significantly higher DSB concentrations, suggesting that the DSB inhibitory activity on Gag assembly was not as strictly sequence-dependent as the negative effect on Gag processing at the CA-SP1 junction [8]. In addition, we found a lower stability of delipidated cores assembled in the presence of DSB, compared to control cores, suggesting a weakening of Gag-Gag interaction occurring in the presence of DSB [14]. Using Gag mutants and a chimeric HIV-MuLV Gag precursor, we mapped the DSB-responsive domain in terms of Gag assembly to the hinge region overlapping the C-terminal end of the CAp24 and the SP1 domain [14].

The DSB concentration at which we observed an inhibitory activity on Gag assembly in insect cells (IC50 ~8–10 μM) was apparently disproportionate compared to the usual doses required for blocking the CAp25 cleavage in HIV-1-infected mammalian cells. However, a wide range of IC-50 values have been reported for the DSB inhibition of virus maturation, varying from nanomolar (0.35 nM [15] and 7.8 nM [2]) to micromolar values (10 μM [12]), depending on the different assays used. In addition, in Pr55Gag-expressing Sf9 cells, the bulk of Gag protein molecules synthesized at 48 h pi has been evaluated to be as high as 5 × 10^8 ^per cell [16]. The addition of DSB at 10 μg/ml to 10^6 ^cells corresponded to 12 × 10^9 ^DSB molecules per cell, i.e. a DSB to Gag stoichiometric ratio of 24: 1 at this DSB concentration. A 24-fold excess of DSB over Gag was therefore compatible with a mechanism of Gag assembly inhibition due to a stoichiometric interaction between the drug and its protein target.

Whatever the molecular mechanism, our observation raised the question of the difference between Pr55Gag-expressing Sf9 cells, in which DSB inhibited VLP assembly [14], versus HIV-1-infected human cells, in which DSB was found to block the CA-SP1 (CAp25) to CAp24 maturation cleavage [3,4,8,11], and to have limited effects on virus assembly [1]. In our experimental model of baculovirus-infected cells [14], assembly of Pr55Gag was analyzed in a context devoid of PR and of glycoproteins (Gp) SUgp120 and TMgp41, three viral components which have been identified as directly or indirectly involved in the antiviral effects of betulinic acid derivatives [8,17,18]. In the aim to reconcile the different antiviral activities of DSB, we explored cellular and viral determinants of the DSB response, and their possible role in modulating the degree of susceptibility to DSB of the VLP assembly process. Among the viral candidates, we analyzed EnvGp160, the precursor to the envelope glycoproteins (reviewed in [19]), and two inner core components, the Vpr and Vif proteins. Vpr is packaged into the virion in substoichiometric amounts with Gag [20-23], and Vif, which is also coencapsidated with Gag, has been found to exert a control on proteolytic processing of Gag in insect cells [24] and human cells [25].

We found that coexpression of wild-type Vif protein (Vif^wt^) with Pr55Gag restored the VLP assembly in DSB-treated Sf9 cells at levels observed in the absence of the drug, suggesting an antagonistic effect of Vif towards DSB. Data obtained with Vif mutants indicated that the anti-DSB function of Vif required the integrity of the zinc binding domain (ZBD) recently identified in the Vif protein [26-28], but was independent of the Vif packaging function. Electron microscopic analysis showed that coexpression of Pr55Gag and Vif^wt^, in the presence or absence of DSB, resulted in a major change in the VLP egress pathway: the majority of VLP budded in intracytoplasmic vesicles and were released by exocytosis, instead of budding at the plasma membrane as in cells expressing Pr55Gag alone. With ZBD mutants of Vif however, the VLP budding pathway was similar to that observed in cells expressing Pr55Gag alone. Our data suggested that the anti-DSB effect of Vif, a novel function associated with its ZBD, was the indirect consequence of its effect on the cellular pathway of VLP assembly and budding.

## Results

### Antiviral effects of DSB and cellular context

We first compared the effect of DSB on VLP assembly and release in our reference model of AcMNPV-Pr55Gag-infected Sf9 cells [14] and in a *trans*-packaging mammalian cell line. 5BD.1 cells derive from CMT3-COS cells by integration of a discontinuous HIV-1 progenome, and stably express the *gag*, *gagpol*, *rev *and *env *gene products but no Nef protein. 5BD.1 cells also express Vif protein in significant amounts [29,30]. 5BD.1 and Sf9 cells represented a similar situation in terms of VLP content, as both cell types produced VLP devoid of viral genomic RNA. DSB was added to monolayers of 5BD.1 cells at increasing concentrations for 30 h, and whole cell lysates and VLP recovered from culture medium were analyzed for Gag protein content at the end of this time period.

**Figure 1:**
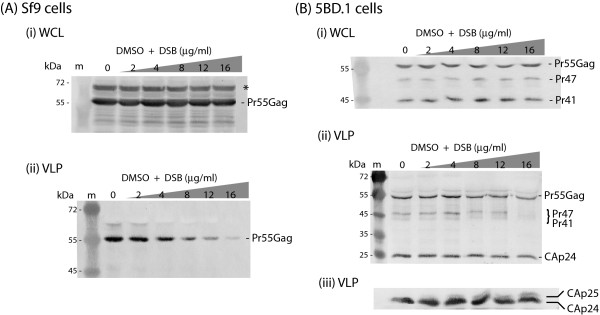
**Effects of DSB on HIV-1 VLP production by (A) insect cells and (B) mammalian cells**. **(A)**, Sf9 cells infected with AcMNPV-Pr55Gag were treated with increasing concentrations of DSB in DMSO-aliquots for 30h at 18h pi, as indicated on top of the panels. Cells were harvested at 48 h pi, and whole cell lysates (WCL) and extracellular VLP recovered from the culture medium were analyzed by SDS-PAGE and immunoblotting using anti-Gag polyclonal antibody and phosphatase-labelled anti-rabbit IgG antibody. **(i)**, WCL. (*), Asterisk marks posttranslationally modified Gag precursor (ubiquitinated and/or phosphorylated). This Gag species was not included in the quantification of Pr55Gag polyprotein. **(ii)**, Extracellular VLP. (**B**), 5BD.1 packaging cells were treated with increasing DSB concentrations in DMSO for 30 h, as indicated on top of the panels, and cells and VLP collected separately and analyzed as above. **(i)**, WCL; **(ii)**, VLP. **(iii)**, Same experiment as in (**ii**), except for the immunoblot analysis, which was performed using ^35^S-labelled secondary antibody. Shown in (**iii**) is an autoradiogram of the blot. Molecular markers (m) were electrophoresed on the left side of the gels, and their molecular masses are indicated in kiloDaltons (kDa).

The intracellular Gag content was found to remain constant throughout the period of DSB treatment in both Sf9 and 5BD.1 cells (Fig. 1Ai and 1Bi), which confirmed that DSB had no significant effect on the level of Gag protein synthesis [14]. However, a drastic decrease in the yields of extracellular VLP was observed at DSB doses superior to 4 μg/ml in Pr55Gag-expressing Sf9 cells (Fig. 1Aii; and refer to [14]). By contrast, only a moderate decrease in VLP production (20–30%) was detected for DSB-treated 5BD.1 cells at high DSB concentrations (12 to 16 μg/ml; Fig. 1Bii). Protein analysis of VLP showed that their Gag protein content mainly consisted of Pr55Gag and CAp24 proteins, with other minor species migrating at the expected position for intermediate cleavage products, e.g. Pr47 to Pr41 (Fig. 1Bii). Prolonged exposure of autoradiograms of immunoblots reacted with radiolabelled secondary antibody revealed a discrete alteration of the Gag processing at high DSB concentrations: there was a progressive increase in the amount of uncleaved CAp25 versus the CAp24 species (Fig. 1Biii), as expected from previous studies [3,4,8,11].

VLP assembly and release were therefore less sensitive to DSB inhibitor in 5BD.1 cells compared to Gag-expressing Sf9 cells. This suggested that the DSB sensitivity of the VLP assembly pathway might be modulated by the cellular context in which the HIV-1 Gag precursor was expressed, or/and by viral proteins present in 5BD.1 cells and absent from Sf9 cells. The following experiments were designed to address this issue, and to determine which factor(s) possibly interfered with DSB inhibitory activity and accounted for the difference in DSB response between Sf9 and 5BD.1 cells, as well as other mammalian cells.

### Absence of detectable effect of EnvGp160 or Vpr on the DSB inhibition of VLP assembly in Sf9 cells

The best candidates to act as viral modulators of the Gag assembly response to DSB were the HIV-1 proteins coencapsidated with Gag, in particular those which are active participants in the virus assembly pathway (reviewed in [19,31]). This was the case for the envelope glycoprotein Gp160, which has been shown to interact with the MA protein via the cytoplasmic tail of its TMgp41 domain [32-36], as well as for auxiliary viral proteins Nef, Vpr and Vif.

In order to test this possibility, Sf9 were coinfected with AcMNPV-Pr55Gag and AcMNPV-Gp160, and subjected to increasing doses of DSB for 30 h, at 18 h pi. Culture medium samples were collected at 48 h pi and assayed for production of extracellular VLP. Results were compared with VLP yields from Sf9 cells infected with AcMNPV-Pr55Gag alone and treated in parallel with DSB at the same doses. No significant difference in the DSB effect on VLP assembly was detectable with or without coexpression of EnvGp160 (data not shown). This excluded the direct or indirect participation of HIV-1 envelope glycoproteins in the level of susceptibility to DSB of assembly and extracellular release of VLP by Sf9 cells.

Nef in its processed form, called Nef core, has been shown to be a *bona fide *component of the virion inner core [37-40]. In 5BD.1 cells, which do not express Nef but express Vif [29,30], we observed a significantly lesser inhibitory effect of DSB on VLP assembly, compared to Gag-expressing Sf9 cells (refer to Fig. 1Bii). Considering that Nef protein was absent from both Sf9 and 5BD.1 cells, the difference in DSB response between these two cell types apparently excluded Nef as a possible modulator of the DSB sensitivity of VLP assembly.

**Figure 2:**
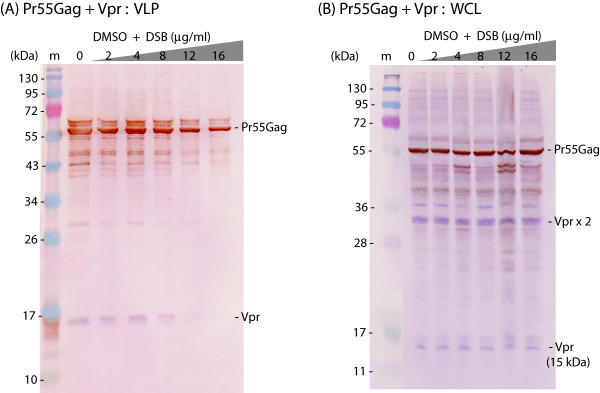
**Absence of counteracting effect of Vpr on DSB inhibition of HIV-1 VLP assembly and release**. Sf9 cells were coinfected with two baculoviruses at equal MOI each (5 PFU/cell), one expressing Pr55Gag, the other expressing His-tagged Vpr. Cells were treated with increasing concentrations of DSB in DMSO aliquots for 30 h at 18 h pi, as indicated on top of the panels. Cells were harvested at 48 h pi, and whole cell lysates (WCL) and extracellular VLP analyzed by SDS-PAGE and immunoblotting, using anti-His mAb and phosphatase-labelled anti-mouse IgG antibody, followed by anti-Gag rabbit antibody and peroxidase-labelled anti-rabbit IgG antibody. **(A)**, VLP. **(B)**, WCL. Note the occurrence of Vpr dimer (Vprx2; 30 kDa), stained in blue with the phosphatase reaction. (m), prestained molecular mass markers; (kDa), kiloDaltons.

Vpr is coencapsidated with Gag via interaction of the N-terminal alpha-helical domain encompassing residues 17–33 in Vpr [41-44] with the LXXLFG motif in the p6 domain of Gag [21,22,45-48]. In Sf9 coinfected with AcMNPV-Pr55Gag and AcMNPV-Vpr, the same DSB sensitivity of VLP assembly was observed as in cells solely expressing AcMNPV-Pr55Gag: both Pr55Gag and Vpr protein signals decreased in parallel and in DSB dose-dependent manner in the extracellular medium of DSB-treated cells, although their intracellular content remained unchanged (Fig. 2). This implied that Vpr did not significantly interfere with the inhibitory effect of DSB on Gag assembly.

### Antagonistic effect of Vif^wt ^on the DSB inhibition of HIV-1 VLP assembly

**Figure 3:**
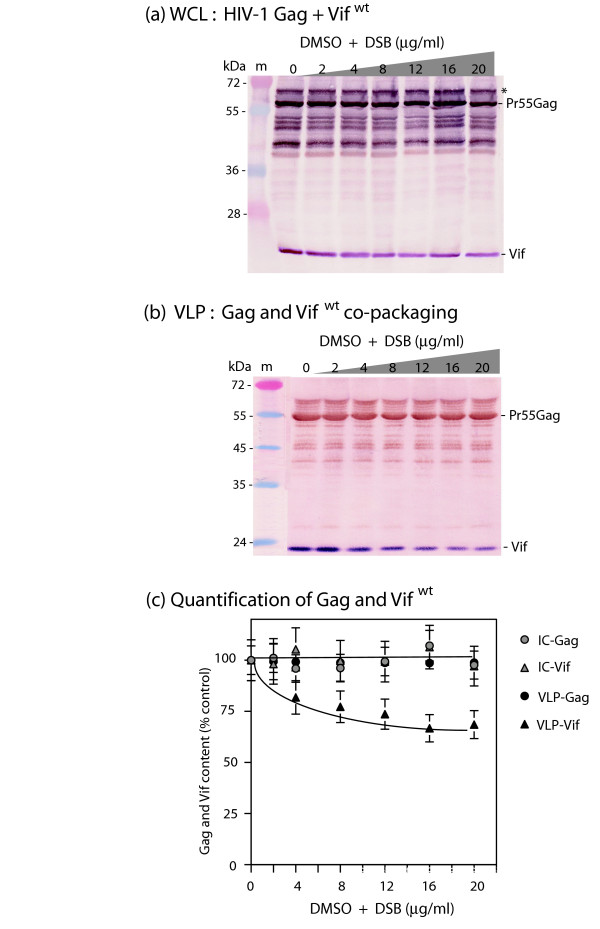
**Influence of Vif on the DSB susceptibility of HIV-1 VLP assembly in Sf9 cells**. Sf9 cells were coinfected with equal MOI (5 PFU/cell) of two baculoviruses expressing Pr55Gag and Vif, respectively. Cells were treated with increasing concentrations of DSB in DMSO for 30 h at 18 h pi, as indicated on top of panels (a) and (b), and the *x*-axis of panel (c). Cells were harvested at 48 h pi, and whole cell lysates (WCL) and extracellular VLP analyzed by SDS-PAGE and immunoblotting. Blots were reacted with anti-Vif primary antibody and secondary phosphatase-labelled antibody, followed by anti-Gag primary antibody and secondary peroxidase-labelled antibody. **(a)**, WCL. (*), Asterisk marks posttranslationally modified Gag precursor (ubiquitinated and/or phosphorylated). This Gag species was not included in the quantification of Pr55Gag polyprotein. **(b)**, VLP. Molecular mass of prestained markers (m) are indicated in kiloDaltons (kDa) on the left side of panels (a) and (b). **(c)**, Quantification of Gag and Vif proteins in WCL (IC-Gag, intracellular Gag; IC-Vif, intracellular Vif) and extracellular VLP, using SDS-PAGE and radio-immunoblotting. Gag and Vif protein contents were quantified by autoradiography of immunoblots reacted with anti-Gag and anti-Vif rabbit primary antibodies and ^35^S-labelled secondary anti-rabbit IgG antibody. After autoradiography of the blots, bands of Pr55Gag and Vif proteins were excised and their radioactive content determined by liquid scintillation spectrometry. Results were expressed as percentage of control, untreated samples, which was attributed the 100% value. Mean of three separate experiments ± standard deviation.

HIV-1 Vif protein has been shown to interact with Pr55Gag *in vitro *and *in vivo *[49,50], to control the viral PR-mediated processing of Gag in mammalian and insect cells [24,25,51], and to be coencapsidated with Gag at a level of 70–100 copies of Vif protein per HIV-1 virion or VLP [24,25,50,52-57]. Sf9 cells coinfected with AcMNPV-Pr55Gag and AcMNPV-Vif^wt ^showed a pattern of DSB effect different from that observed in cells expressing Pr55Gag alone: there was no significant decrease in the VLP yields from DSB-treated Sf9 cells, up to drug concentrations as high as 20 μg/ml, implying that expression of Vif^wt ^protein negated the DSB inhibition of VLP assembly process in Pr55Gag-expressing insect cells (Fig. 3b, c). Of note, the Vif content of VLP progressively decreased in a DSB-dependent manner (25–30% less than in control sample at 20 μg/ml DSB; Fig. 3b, c), although the intracellular content of Vif and Pr55Gag remained stable up to high DSB doses (16–20 μg/ml; Fig. 3a). This suggested a direct or indirect interference of Vif with DSB in virus assembly, resulting in the abrogation of the DSB negative effect on this process.

### Anti-DSB activity of packaging-defective mutants of Vif

**Figure 4:**
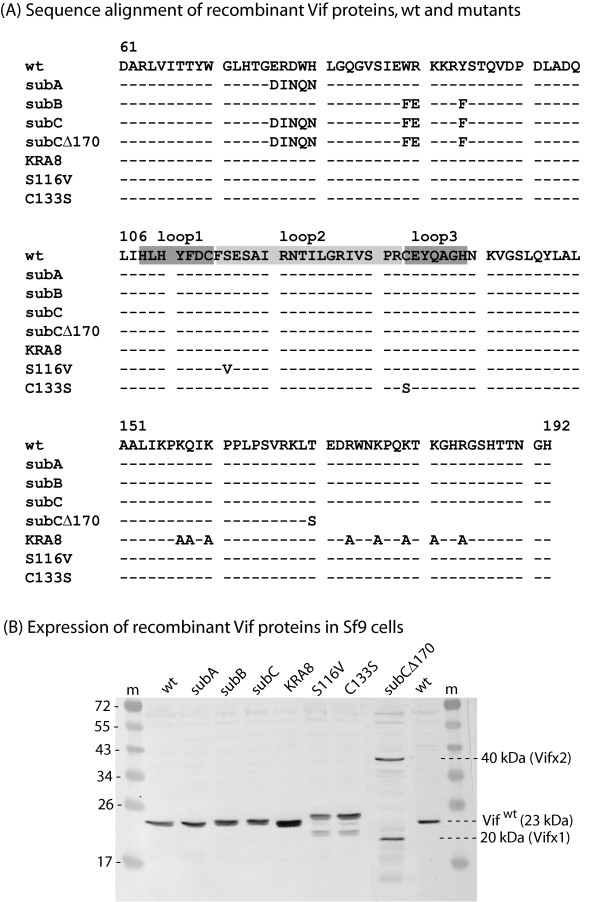
**Genotype and expression of recombinant Vif mutants in Sf9 cells**. **(A)**, Sequence alignment of the central and C-terminal domains of HIV-1 Vif proteins, WT and mutants. The zinc binding domain (ZBD) and its three constitutive loops are boxed: loops 1 and 3 are indicated as dark grey boxes, central loop 2 as a lighter grey box. **(B)**, Cellular expression of recombinant Vif proteins, wild-type and mutants, in baculovirus-infected Sf9 cells. Sf9 cells were infected with baculoviruses (MOI 5) expressing different forms of Vif, as indicated on top of the panel, and harvested at 48 h pi. Whole cell lysates were analyzed by SDS-PAGE and immunoblotting, using anti-Vif primary antibody and secondary peroxidase-labelled antibody. The full-length ZBD mutants VifC133S and Vif116V show an aberrant electrophoretic mobility, as they migrate with a higher apparent molecular weight compared to Vif^wt ^(23 kDa), and a higher sensitivity to proteolysis, as evidenced by the discrete bands of lower molecular weight breakdown products. Note the propensity of the Vif protein of triple mutant Vif*sub*CΔ170 (20 kDa) to dimerize (Vifx2; 40 kDa).

In a previous study, we have constructed and characterized Vif mutants which differed from Vif^wt ^in their efficiency of copackaging with Pr55Gag into VLP produced by recombinant baculovirus-coinfected cells [50]. The two discrete regions involved in this function spanned residues 76–80 and 89–94, respectively (Fig. 4). Substitution mutants Vif*sub*A (^76^EKEWH^80 ^to ^76^DINQN^80^), Vif*sub*B (^89^WR^90^-Y^94 ^to ^89^FE^90^-F^94^), double mutant Vif*sub*C (*sub*A+*sub*B), and triple mutant Vif*sub*CΔ170 carrying the double mutation *sub*A+*sub*B and a deletion of the C-terminal twenty-three residues, were found to be defective to various degrees in the encapsidation of Vif into VLP: Vif*sub*A, Vif*sub*B and Vif*sub*C were partially defective in Vif packaging (40–50% the levels of Vif^wt^), whereas this function was totally abolished in Vif*sub*CΔ170 [50]. On the opposite, VifKRA8, a full-length Vif mutant which had eight basic residues in the C-terminal domain replaced by neutral alanine residues (Fig. 4) and lacked the plasma membrane addressing function [54], was packaged into VLP at levels higher than Vif^wt ^[50], suggesting that plasma membrane localization and encapsidation into VLP were distinct functions in Vif.

**Figure 5:**
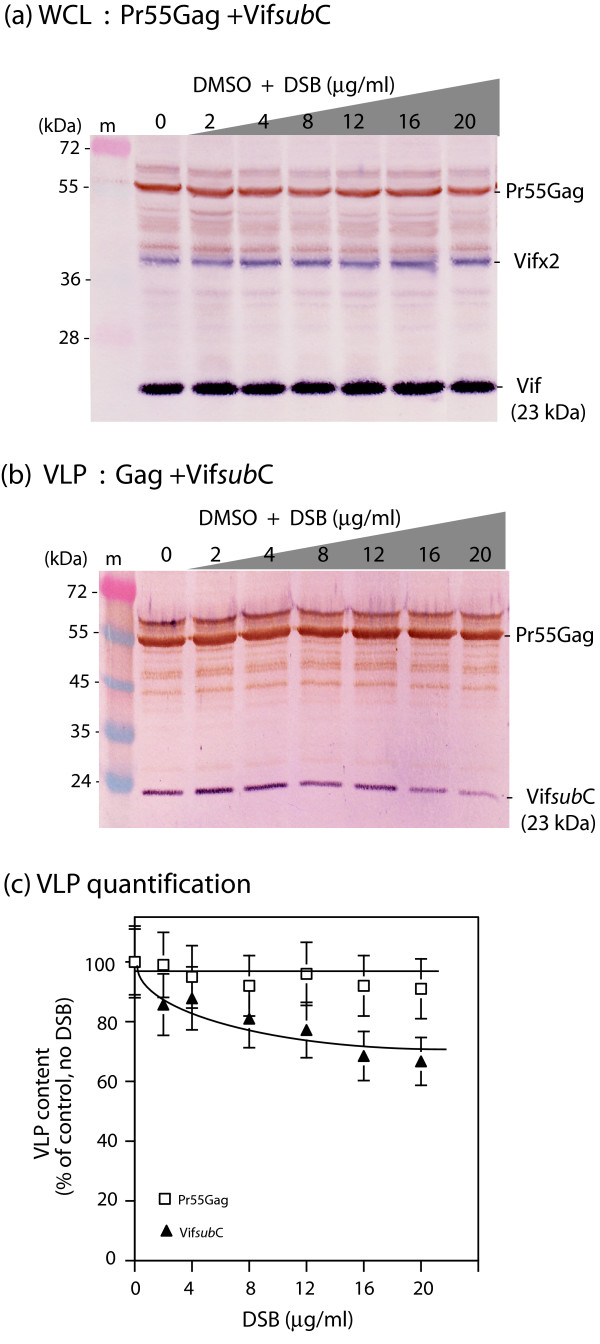
**Counteracting effect of packaging-defective mutant Vif *sub*C on the DSB inhibition of HIV-1 VLP assembly**. Sf9 cells were coinfected with two baculoviruses at equal MOI of each (5 PFU/cell), one expressing Pr55Gag, the other expressing the double substitution, packaging-defective mutant Vif*sub*C. Cells were treated with increasing concentrations of DSB in DMSO for 30 h at 18 h pi, as indicated on top of panels (a) and (b), and on the *x*-axis of panel (c). Cells were harvested at 48 h pi, and whole cell lysates (WCL) and extracellular VLP analyzed by SDS-PAGE and immunoblotting, using anti-Vif primary antibody and secondary phosphatase-labelled antibody, followed by anti-Gag primary antibody and secondary peroxidase-labelled antibody. **(a)**, WCL; **(b)**, VLP. Note the low level of Vif protein in VLP, consistent with the packaging-defective phenotype of Vif*sub*C [50]. (m), prestained molecular mass markers; (kDa), kiloDaltons. **(c)**, Quantification of Pr55Gag and Vif protein content of VLP, performed by autoradiography of immunoblots with anti-Gag and anti-Vif rabbit antibodies and ^35^S-labelled secondary anti-rabbit IgG antibody, as described in the legend to Fig. 3 (c). Results were expressed as percentage of control, untreated samples, which was attributed the 100% value. Mean of three separate experiments ± standard deviation.

We then tested the anti-DSB activity of Vif mutants with different encapsidation phenotypes. With Vif*sub*C, the production of extracellular VLP remained virtually unchanged throughout the DSB concentration range, with less than 15% decrease in VLP production at high DSB doses (Fig. 5). As observed with Vif^wt ^(refer to Fig. 3b, c), there was a DSB-dependent, progressive decrease of Vif*sub*C mutant protein content in VLP, relative to the Pr55Gag content, with 20–30% lesser Vif protein incorporated at high DSB doses, compared to control samples (Fig. 5b, c, samples 16–20). A similar DSB resistance pattern as with Vif^wt ^and Vif*sub*C was observed with the other packaging-defective mutants Vif*sub*A, Vif*sub*B, and Vif*sub*CΔ170 (not shown). Likewise, the packaging-efficient mutant VifKRA8 showed the same phenotype as Vif^wt ^and the packaging-defecting mutants in terms of anti-DSB activity (not shown). These results suggested that the DSB-counteracting function of Vif was independent from the packaging function of Vif.

### Involvement of the zinc-binding domain of Vif in its anti-DSB function

A conserved region of the Vif protein, within residues 108 to 140, has been recently characterized as a non-canonical zinc-coordinating structure, generated by the H^108^, C^114^, C^133 ^and H^139 ^coordinates (HCCH) with a Zn atom [27,28]. This zinc-binding domain (ZBD) has been identified as the interacting region with the Cullin5 (Cul5) E3-ubiquitin ligase [28]. It has been shown that Vif recruits cellular proteins ElonginB/ElonginC and Cul5 via its BC-box and ZBD domain, respectively, and the resulting E3-ubiquitin ligase complex polyubiquitinates APOBEC3G and redirects it to the proteasome [27,28,58-60]. Position 116 in HIV-1 Vif belongs to the ZBD domain, and more precisely to the N-terminal portion of loop 2, the large loop defined by the two cysteine residues at positions 114 and 133 [26,28] (Fig. 4A). It has been recently found that replacement of Ser by Ala at position 116 in Vif did not change the Vif-Cul5 interaction [28]. This result was not totally surprising since position 116 can be occupied by serine, threonine or alanine in HIV-1 and SIV-CPZ strains [61], all residues characterized by short, hydrophilic or hydrophobic, side chains. However, these authors observed that deletion of Ser-116 abolished the Vif-Cul5 interaction, implying that the amino acid residue spacing in loop 2 was critical for Vif functions [28].

**Figure 6:**
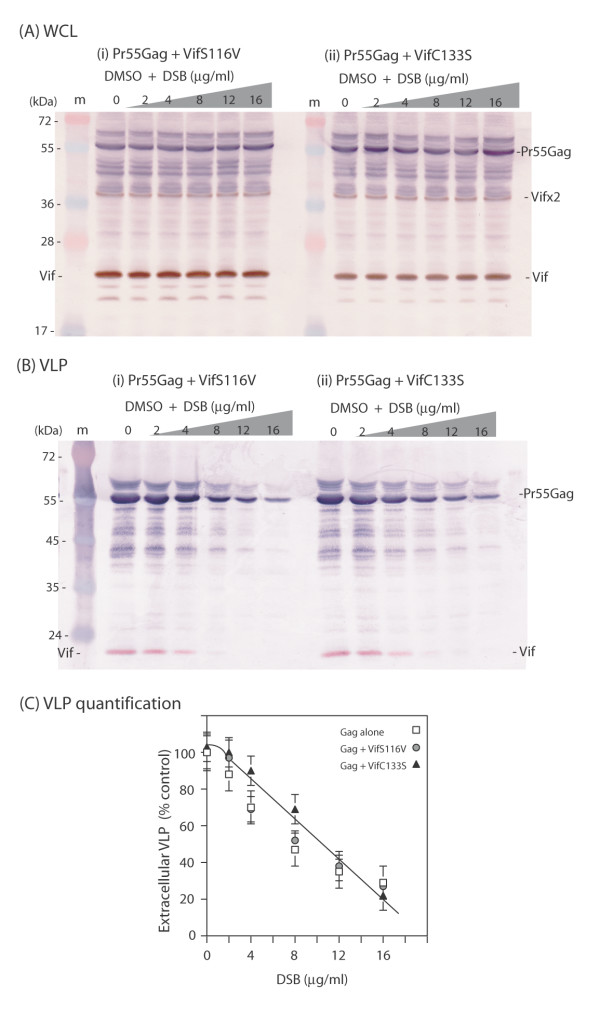
**Absence of anti-DSB effect of zinc-binding domain mutants of Vif**. Sf9 cells were coinfected with two baculoviruses at equal MOI of each (5 PFU/cell), one expressing Pr55Gag, the other expressing VifS116V **(A and B, (i)) **or VifC133S **(A and B, (ii))**. Cells were treated with increasing concentrations of DSB in DMSO for 30 h at 18 h pi, as indicated on top of panels (i) and (ii), and on the *x*-axis of panel (C). Cells were harvested at 48 h pi, and whole cell lysates (WCL) and extracellular VLP analyzed by SDS-PAGE and immunoblotting, using anti-Vif primary antibody and secondary peroxidase-labelled antibody, followed by anti-Gag primary antibody and phosphatase-labelled secondary antibody. **(A)**, WCL; **(B)**, VLP. (m), prestained molecular mass markers; (kDa), kiloDaltons. **(C)**, Quantification of VLP produced by DSB-treated Sf9 cells coexpressing Pr55Gag and Vif mutants was performed using SDS-PAGE and autoradiography of immunoblots reacted with anti-Gag and ^35^S-labelled secondary anti-rabbit IgG antibody, as described in the legends to Fig. 3(c) and 5(c). Results were expressed as percentage of control, untreated samples, which was attributed the 100% value. Mean of three separate experiments ± standard deviation.

Taking the latter observation into account, we substituted the serine residue to a valine at position 116. We assumed that the bulky side chain of valine would introduce local disorganization in the 3D structure of the ZBD domain, as did the S116 deletion, and would be detrimental to the anti-DSB effect of Vif. We found that the VifS116V mutant was coencapsidated with Gag at the same levels as Vif^wt ^(Fig. 6Bi, lane 0). However, the assembly and extracellular release of VLP from Sf9 cells coexpressing Pr55Gag and VifS116V showed the same degree of DSB susceptibility as the one observed when Pr55Gag was expressed alone (Fig. 6Bi, and Fig. 6C). Thus, the lack of antagonistic effect against DSB of the packageable mutant VifS116V confirmed that anti-DSB function and packaging into VLP were separate functions in the Vif protein.

To further analyze the role of the ZBD structure in the Vif anti-DSB activity, we constructed another mutant of recombinant Vif protein. Cysteine at position 133 in Vif is a residue essential for virus infectivity [62,63], for Zn coordinate formation and ZBD-associated functions in Vif [27,28]. We therefore generated mutation C133S in recombinant Vif, and tested mutant VifC133S in co-expression with Pr55Gag in control or DSB-treated Sf9 cells, as above. In untreated cells, VifC133S behaved as VifS116V mutant, and was coencapsidated with Pr55Gag into VLP at levels equivalent to Vif^wt ^(Fig. 6Bii, lane 0). In DSB-treated cell samples, VifC133S had the same phenotype as VifS116V in terms of lack of anti-DSB effect: assembly and release of VLP from Sf9 cells coexpressing Pr55Gag and VifC133S showed the same degree of DSB sensitivity as from Sf9 cells expressing Pr55Gag alone (Fig. 6Bii, and Fig. 6C).

These results suggested that the antagonistic activity of Vif against the DSB inhibition of Gag assembly, absent from VifS116V and VifC133S mutants, was associated with the ZBD and more precisely involved residues located on the N-terminal side of loop 2. Thus, the phenotype of our Vif mutants with respect to their packaging and anti-DSB properties showed that the integrity of the ZBD structure was not required for the packaging of Vif into VLP produced by Sf9 cells, but was crucial for its DSB counteracting effect.

### Assembly and budding pathways of HIV-1 VLP in Vif-expressing Sf9 cells

**Figure 7:**
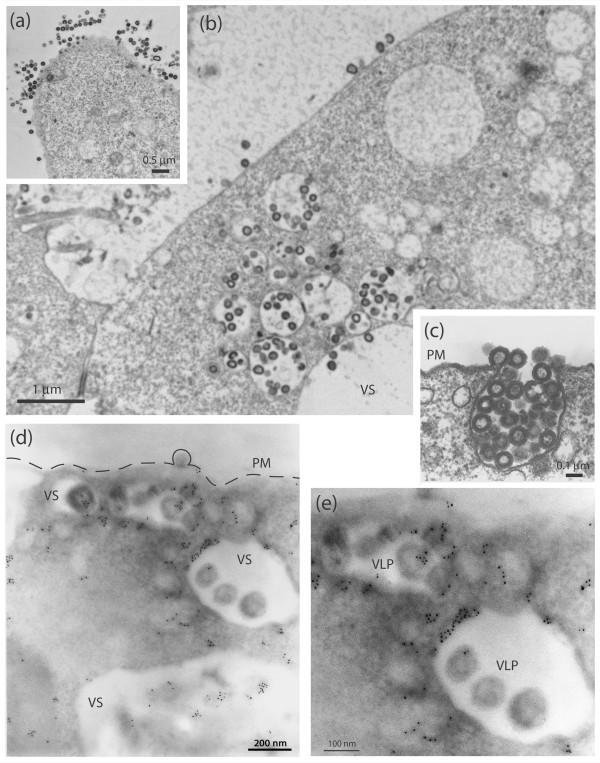
**EM and immuno-EM analysis of Pr55Gag-expressing Sf9 cells, with or without Vif^wt ^coexpression**. Sf9 cells were infected with AcMNPV-Pr55Gag alone or coinfected with another baculovirus expressing Vif (AcMNPV-Vif^wt^) at equal MOI of each (5 PFU/cell), harvested at 48 h pi, and processed for EM analysis. **(a)**, Control cells expressing Pr55Gag alone; **(b)**, Sf9 coinfected with AcMNPV-Pr55Gag and AcMNPV-Vif^wt^. Inset **(c)**, Enlargement of an area of the plasma membrane showing exocytosis of VLP. Note the abundance of VLP at the cell surface in (a), compared to the high VLP content of vesicular compartment in (b). **(d, e)**, Sf9 coinfected with AcMNPV-Pr55Gag and AcMNPV-Vif^wt ^and harvested at 48 h pi were processed for immuno-EM. Cell sections were incubated with anti-Vif rabbit antibody, followed by 5-nm colloidal gold-tagged anti-rabbit IgG antibody. **(d)**, General view of a cell. The plasma membrane (PM) is materialized by a dotted line; the cytoplasmic area shows vesicles (VS) with intraluminal budding of VLP. **(e)**, Enlargement of VLP-containing vesicles. Note the immunogold labelling of VLP, as well as the accumulation of gold grains at the membrane of VLP-containing vesicles.

To further investigate on the mechanism of the DSB counteracting effect of Vif, Sf9 cells coexpressing Pr55Gag and Vif^wt ^or ZBD mutants were analyzed by electron microscopy (EM) and immunoelectron microscopy (immuno-EM). Cells were infected with AcMNPV-Pr55Gag and AcMNPV-Vif, untreated or treated with DSB at 10 μg/ml at 18 h pi, harvested at 48 h pi and processed for EM or immuno-EM using anti-Vif antibody. In control Sf9 cells expressing Pr55Gag alone, the vast majority of VLP assembled at and budded from the plasma membrane (Fig. 7a), as shown in previous studies [16,64,65]. The pattern of VLP assembly and budding was drastically different in Gag+Vif^wt^-coexpressing cells: VLP were found in abundance in cytoplasmic vesicles (Fig. 7b). Coexpression of Vif^wt ^did not decrease the production of VLP by Pr55Gag-expressing Sf9 cells [24,50], and vesicular VLP egressed into the extracellular medium by exocytosis (Fig. 7c). In immuno-EM, gold grains of anti-Vif antibodies were seen in close association with intravesicular VLP, or along the rim of VLP-containing vesicles (Fig. 7d, e), suggesting that Vif and Pr55Gag proteins colocalized in the same vesicular compartment.

**Figure 8:**
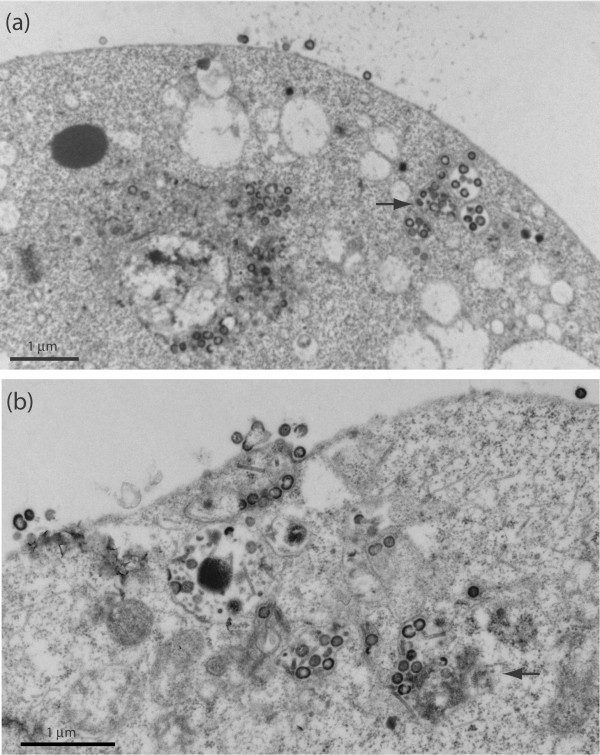
**DSB treatment of Sf9 cells coexpressing Pr55Gag and Vif^wt^**. Sf9 coinfected with AcMNPV-Pr55Gag and AcMNPV-Vif^wt^at equal MOI of each (5 PFU/cell) were treated with DSB at 10 μg/ml for 30 h at 18 h pi. Cells were harvested at 48 h pi, and processed for EM. **(a)**, General view of a cell. **(b)**, Enlargement of a submembranal region of the cell showing VLP in the process of exocytosis. Note the abundance of VLP in the vesicular compartment in panels (a) and (b). VLP-containing vesicles reminiscent of MVBs observed in mammalian cells are indicated with arrows.

The proportion of VLP following the intravesicular budding and exocytosis pathway compared to the ones using the plasma membrane pathway was estimated under the EM, by counting several hundreds of VLP in subcellular compartments of more than 20 different cells. In control Sf9 cells expressing Pr55Gag alone, less than 5% VLP were found within the vesicular compartment, whereas in Gag+Vif^wt^-coexpressing cells, the proportion increased to 30 to 50%, viz. a 5- to 10-fold increase. Likewise, in cells coexpressing Pr55Gag and Vif^wt ^and treated with DSB, most VLP used the intravesicular budding and exocytic pathway (Fig. 8). Interestingly, many VLP-containing vesicles showed an electron-dense, heterogenous lumen (Fig. 8), resembling multivesicular bodies (MVBs) observed in mammalian cells. MVBs belong to the late endosomal subcellular compartment, and have been identified as the preferred budding sites for WT HIV-1 particles in primary human macrophages (reviewed in [66]), as well as in human epithelial and T cells for *gag *mutants altered in the cluster of basic amino acids of the matrix (MAp17) domain [67].

**Figure 9:**
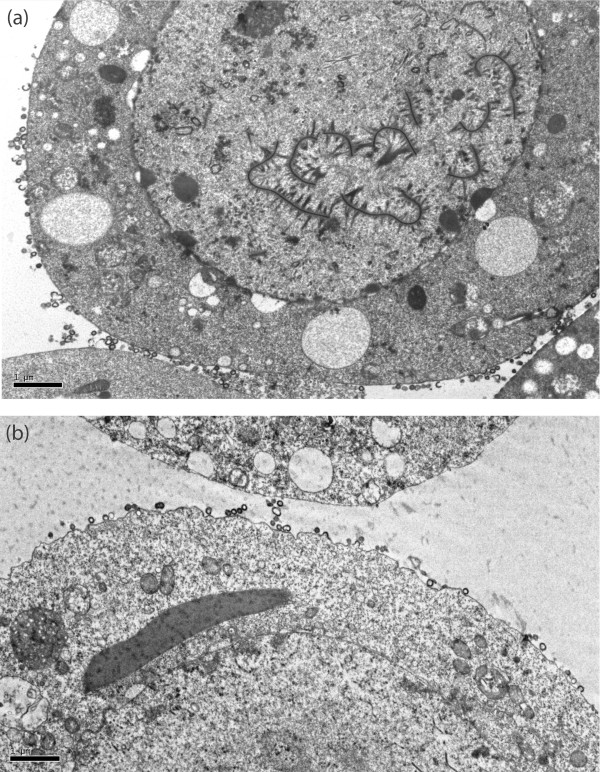
**EM analysis of Sf9 cells coexpressing Pr55Gag and ZBD mutants of Vif**. Sf9 were coinfected with AcMNPV-Pr55Gag and AcMNPV-VifS116V (**a**) or AcMNPV-VifC133S (**b**) at equal MOI of each (5 PFU/cell), harvested at 48 h pi, and processed for EM analysis. The vast majority of VLP budding at the plasma membrane was reminiscent of Sf9 cells expressing Pr55Gag alone (refer to Fig. 7a), and contrasted with Sf9 cells coexpressing Pr55Gag and Vif^wt ^(refer to Fig. 7b-e).

We next examined cells coexpressing Pr55Gag and ZBD mutants of Vif under the EM, and found that, in the presence of Vif116V and VifC133S, the VLP budding pathway was similar to the one observed in Sf9 cells expressing Pr55Gag alone, i.e. a majority of VLP budding at the plasma membrane and rare intravesicular VLP (less than 10%; Fig. 9). The EM pattern of VifS116V and VifC133S mutants was consistent with their phenotype, as both mutants failed to negate the inhibitory effect of DSB on VLP assembly. Taken together, our results suggested that, in the presence of Vif^wt^, the VLP assembly and budding process was redirected to the vesicular compartment, and that the VLP egress via exocytosis represented a salvage pathway through which HIV-1 VLP escaped the negative effect of DSB.

## Discussion

It is generally accepted that DSB inhibits the cleavage of CAp25 into CAp24 and SP1 by the viral PR, due to its interference with the Gag substrate [8]. However, in recombinant Pr55Gag-expressing Sf9 cells, a cellular context devoid of PR and other viral proteins, DSB showed a dose-dependent inhibitory activity on VLP assembly and release [14]. The aim of the present study was to understand this dual inhibitory activity, and explain the apparent discrepancy between the DSB effects observed in mammalian and non-mammalian, insect cells. We first explored the effect of DSB on VLP production in 5BD.1 cells, a mammalian *trans*-packaging cell line producing VLP devoid of viral genome, as the VLP produced by AcMNPV-Pr55Gag-infected Sf9 cells. We found that DSB had only a moderate inhibitory effect on VLP yields at high DSB doses (Fig. 1), indicating that VLP assembly in 5BD.1 cells was less sensitive to DSB inhibitor, compared to Pr55Gag-expressing Sf9 cells. This suggested that the DSB negative effect on the VLP assembly process might be modulated by factors depending on the cellular or/and viral context.

We therefore investigated on the possible influence of viral components on the pattern of anti-assembly effect of DSB, and in particular the role of viral partners of Pr55Gag within the capsid. Coexpression of recombinant Pr55Gag with EnvGp160 or Vpr did not modify the level of inhibition of VLP assembly by DSB (Fig. 2), whereas coexpression of Vif^wt ^restored the production of VLP in DSB-treated cells to levels found in the absence of the drug (Fig. 3). A panel of recombinant Vif mutants (Fig. 4) were then tested for their anti-DSB activity. We found that the DSB-antagonistic effect of Vif was retained in packaging-defective mutants of Vif (Fig. 5), but abolished by a Cys-to-Ser substitution at position 133 (Fig. 6Bii), a mutation which destroyed the zinc finger-like structure or ZBD. A phenotype similar to that of VifC133S was observed for mutant VifS116V (Fig. 6Bi), which carried a mutation on the N-terminal side of the large loop (loop 2) generated by the four HCCH coordinates with the Zn atom (Fig. 4A). Both VifC133S and VifS116V mutants were encapsidated into VLP at levels comparable to Vif^wt ^(Fig. 6Bi and 6Bii, control lanes 0). Our results therefore suggested that (i) the anti-DSB effect and packaging into VLP were two independent functions in Vif; (ii) the function of Vif which negated the DSB-induced inhibition of VLP assembly depended on the integrity of the zinc-binding domain, and more precisely on a discrete region of loop 2 overlapping residue 116 (Fig. 4A). This region differed from the Vif packaging signals [50].

EM analysis of Sf9 cells coexpressing Gag and Vif^wt ^or Vif mutants gave some insight into the cellular mechanism of anti-DSB activity of Vif. Sf9 cells coexpressing Pr55Gag and Vif^wt^, with or without treatment with inhibitory doses of DSB, showed a high proportion of VLP budding into intracytoplasmic vesicles and egressing via exocytosis (Fig. 7b–e and Fig. 8). This contrasted with cells expressing Pr55Gag alone, in which the majority of VLP budded at the plasma membrane (Fig. 7a). When Pr55Gag was coexpressed with one or the other of the ZBD mutants, VifS116V or VifC133S, we observed a drastic change in VLP budding, compared to Vif^wt ^coexpression, consisting of a reversion to the plasma membrane budding pathway, as in Sf9 cells expressing Pr55Gag alone (Fig. 9). Since both ZBD mutants lacked the anti-DSB activity and failed to redirect VLP to the vesicular compartment, as did Vif^wt^, it might be hypothesized that the antagonistic activity of Vif towards DSB would be the indirect effect of a Vif-mediated change in the VLP assembly sites and mode of cellular exit.

It has been shown that the assembly and release of HIV-1 virions proceeds via two pathways, depending upon the cell type [67]: (i) in primary human macrophages, virions preferentially follow the exosomal pathway via MVBs [67-69]; (ii) in HeLa cells and T lymphocytes, the major exgress route consisted of plasma membrane addressing and direct budding at the cell surface, but MA polybasic signal mutants of Gag use the MVB pathway in these cells [67]. Sf9 cells expressing Pr55Gag alone belonged to the second category of cells [16,64,65], but when coexpressed with Vif^wt^, the VLP assembly and budding process mimicked the MVB budding and exocytic pathway used by MA polybasic mutants in HeLa and T cells. The hypothesis formulated above implied that the intravesicular budding and exocytic pathway of VLP would be less sensitive to DSB inhibitory activity than the plasma membrane assembly and budding pathway usually observed in insect cells. If confirmed, this would be an example of drug resistance mechanism (DSB, in the present case) which involves the bypass of a drug-sensitive assembly and budding pathway by the virus or virus-like particle progeny.

The results of our study suggested that DSB and other betulinic acid derivatives could be considered not only as antivirals for patients treatment in vivo, but also as chemical probes to analyse the molecular and cellular mechanisms of retroviral Gag assembly in vitro. In the latter context, considering Vif as a determinant of the budding pathway usage in Sf9 cells, and as a modulator of the DSB response in terms of VLP assembly, any evaluation of potential HIV-1 assembly inhibitors using the baculovirus-insect cell system should be carried out in the presence of the Vif protein.

## Methods

### Chemical synthesis of DSB

The title compound 3-*O*-(3',3'-dimethylsuccinyl)-betulinic acid (C_36_H_56_O_6_; MW = 584.8) was obtained as originally described [5] with a few minor modifications described in our previous study [14].

### Cells

Simian 5BD.1 packaging cells (obtained from D. Rekosh and M.-L. Hammarskjöld, University of Virginia at Charlottesville) were CMT3-COS-derived cells that stably express HIV-1 Gag-Pol and Env proteins but no Nef [29,30]. They were maintained in Iscove's medium supplemented with bovine calf serum (10%), hygromycin (200 μg/ml), gentamycin (50 μg/ml) and G418 (1.5 mg/ml). *Spodoptera frugiperda *Sf9 cells were maintained as monolayers, and infected with recombinant baculovirus at a multiplicity of infection (MOI) ranging from 2.5 to 20 PFU/cell, as previously described [16,65,70,71].

### Recombinant baculoviruses

All the different HIV-1 genes used in the present study, except for *vpr*, were inserted into the genome of *Autographa californica *MultiCapsid NucleoPolyhedrosis Virus (AcMNPV) under the control of a chimeric AcMNPV-GmNPV polyhedrin promoter [16,65,70]. ***(i) Gag***. AcMNPV-Pr55Gag, expressing the full-length wild type (WT) HIV-1 Gag polyprotein (Pr55Gag), has been described in detail in previous studies [14,16,65,71]. ***(ii) Envelope glycoprotein Gp160***. AcMNPV-Gp160 expressed the CCR5-tropic YU2 envelope glycoprotein. ***(iii) Vpr***. The baculovirus clone expressing the oligohistidine-tagged Vpr protein (AcMNPV-Vpr) was obtained from Eric Cohen [72]. ***(iv) Vif clones ***(refer to Fig. 4). AcMNPV-Vif^wt ^expressed the full-length wild type Vif protein. Vif*sub*A (EKEWH-to-DINQN substitution) and Vif*sub*B (WRxxxY-to-FExxxF substitution) were mutated in two tryptophan-containing motifs, at position 76–80 and 89–94, respectively; the double mutant Vif*sub*C carried both *sub*A and *sub*B mutations; mutant VifKRA8 had 8 basic residues in the C-terminal domain (residues 156–192) replaced by alanine residues. Vif*sub*CΔ170 carried the Vif*sub*C multiple substitutions and an additional deletion of the 23 C-terminal residues of Vif. Vif*sub*A, Vif*sub*B, Vif*sub*C, VifKRA8 and Vif*sub*CΔ170 have been characterized in previous studies [49,50]. Substitutions Ser-to-Val at position 116 and Cys-to-Ser at position 133 in the Vif sequence were constructed using the conventional PCR-SOE technique. Recombinant Vif mutants VifS116V and VifC133S were generated by recombination with the baculoviral genome. All mutants were verified by DNA sequencing.

### Gag assembly assays

Aliquots of Sf9 cells (10^6^) were infected with recombinant AcMNPV at MOI 10. At 18 h postinfection (pi), increasing quantities of DSB in DMSO were added. To avoid possible interference with DMSO effect, DMSO was kept constant in volume in the different samples. A stock solution of DSB (10 mg/ml DMSO) was diluted with DMSO to obtain a range of DSB concentrations from 0.5 to 30 μg DSB per 3 μl-aliquot of DMSO, and each 3 μl-aliquot was added to 1 ml of culture medium overlaying the cell monolayers. The cells were harvested at 48 h pi, and extracellular VLP quantitatively assayed in the culture medium.

### Isolation of extracellular virus-like particles (VLP)

Sf9 cell culture supernatants were clarified by low-speed centrifugation, then VLP recovered using sucrose-step gradient centrifugation[73], by pelleting through a cushion of 20% sucrose in TNE buffer (TNE: 100 mM NaCl, 10 mM Tris-HCl pH 7.4, 1 mM Na_2_EDTA). The pellets were gently resuspended in PBS (0.20–0.25 ml), and VLP further purified by isopycnic ultracentrifugation in linear sucrose-D_2_O gradients [50]. Gradients (10-ml total volume, 30–50%, w:v) were generated from a 50% sucrose solution made in D_2_O buffered to pH 7.2 with NaOH, and a 30% sucrose solution made in 10 mM Tris-HCl, pH 7.2, 150 mM NaCl, 5.7 mM Na_2_EDTA. The gradients were centrifuged for 18 h at 28 krpm in a Beckman SW41 rotor. Aliquots of 0.5 ml were collected from the top, and proteins analyzed by SDS-PAGE, immunoblot analysis with or without autoradiography.

### Gel electrophoresis and membrane transfer

Polyacrylamide gel electrophoresis of SDS-denatured protein samples (SDS-PAGE), and immunoblotting analysis have been described in detail in previous studies [70,71,74]. Briefly, proteins were electrophoresed in SDS-denaturing, 12%-polyacrylamide gel and electrically transferred to nitrocellulose membrane (Hybond™-C-extra; GE Healthcare Bio-Sciences). Blots were blocked in 5% skimmed milk in Tris-buffered saline (TBS) containing 0.05% Tween-20 (TBS-T), rinsed in TBS-T, then successively incubated with primary rabbit, mouse or goat anti-Gag antibodies, and relevant anti-IgG secondary antibodies, at working dilutions ranging from 1:5,000 to 1:40,000. Apparent molecular weights were estimated by comparison with prestained protein markers (PageRuler™ prestained protein ladder; Fermentas Inc., Hanover, MD).

### Antibodies and immunological analysis

Anti-HIV-1 Gag polyclonal antibody (laboratory-made; [50]) was raised in rabbit by injection of bacterially-expressed, GST-fused and affinity-purified C-truncated Gag protein consisting of full-length MA domain and the first seventy-eight residues of the CA domain (*Pst *I site; *gag*_Lai _sequence). Mouse monoclonal antibody (mAb) anti-CAp24 (Epiclone #5001) and mAb anti-MAp17 (Epiclone #5003) were obtained from Cylex Inc. (Columbia, MD). MAb 41A9, directed against the Gp41 domain of the EnvGp160, was obtained from Hybridolab (Institut Pasteur, Paris). Mouse anti-Hisx6-tag antibody (Tag-100 antibody) was purchased from Qiagen SA (Courtabæuf, France). Anti-Vif antibody was raised in rabbit by injection of bacterially-expressed His-tagged Vif protein purified by guanidine denaturation and progressive renaturation of insoluble protein inclusion, followed by affinity chromatography on Ni-column (a gift from E. Decroly; [75]). Phosphatase-labelled anti-rabbit, or anti-mouse IgG conjugates were purchased from Sigma (St Louis, MO), and horseradish peroxidase-labelled conjugates from Sigma (St Louis, MO). For immunological quantification of membrane-transferred Gag and Vif proteins, blots were reacted with secondary ^35^SLR-labelled anti-rabbit or anti-mouse whole IgG antibody (GE Healthcare Bio-Sciences; 2,000 Ci/mmol; 20–30 μCi per 100 cm^2 ^membrane), and exposed to radiographic films (Hyperfilm™ MP, GE Healthcare Bio-Sciences). Autoradiograms were scanned and quantitated by densitometric analysis, using the VersaDoc image analyzer and the Quantity One program (BioRad). Alternatively, protein bands were excised from blots and radioactivity measured in a scintillation counter (Beckman LS-6500), as previously described [14,50].

### Electron microscopy (EM) and immunoelectron microscopy (immuno-EM)

Baculovirus-infected Sf9 cells were harvested at 48 h pi, pelleted, fixed with 2.5% glutaraldehyde in 0.1 M phosphate buffer, pH 7.5, post-fixed with osmium tetroxide (2% in H_2_O) and treated with 0.5% tannic acid solution in H_2_O. The specimens were dehydrated and embedded in Epon (Epon-812; Fulham, Latham, NY). Ultrathin sections were stained with 2.6% alkaline lead citrate and 0.5% uranyl acetate in 50% ethanol, and post-stained with 0.5% uranyl acetate solution in H_2_O [64]. For immuno-EM analyses, cell specimens were included in metacrylate resin (Lowicryl K4M). Sections on grids were reacted with polyclonal anti-Vif antibody (diluted at 1:100 in Tris-buffered saline) overnight at 4°C. Reaction with by 5-nm gold-conjugated anti-rabbit IgG goat antibody (EM-GAR5; British Biocell International, Cardiff, UK) was carried out at room temperature for 1 h, and sections post-stained with 0.5% 0.5% uranyl acetate solution in H_2_O [50,64,74,76]. Grids were examined under a Jeol JEM-1400 electron microscope, equiped with an ORIUS™ digitalized camera (Gatan France, 78113-Grandchamp). For statistical EM analyses, a minimum of 30 grid squares containing 10 to 20 cell sections each were examined for counting VLP budding at the cell surface, or for core-like particles assembled intracellularly.

## Competing interests

The authors declare that they have no competing interests.

## Authors' contributions

SDF performed the bench work, and BG and PB performed the EM analyses. PC synthesized the DSB. SSH and PB conceived the strategies and designed the experiments. SB contributed to data analysis. PB wrote the manuscript. All the authors read and approved the final manuscript.
